# Conventional intramuscular sedatives versus ziprasidone for severe agitation in adolescents: case-control study

**DOI:** 10.1186/1753-2000-3-9

**Published:** 2009-03-12

**Authors:** William C Jangro, Horacio Preval, Robert Southard, Steven G Klotz, Andrew Francis

**Affiliations:** 1 Dept. Psychiatry & Behavioral Sciences, SUNY at Stony Brook, Stony Brook, NY 11794, USA

## Abstract

**Objective:**

The objective of this study was to compare intramuscular (IM) ziprasidone to conventional IM medications (haloperidol combined with lorazepam) for the treatment of severe agitation in adolescents (age 12–17).

**Methods:**

We retrospectively identified consecutive severe agitation episodes (defined as requiring physical restraint) in adolescents treated with either IM ziprasidone or conventional IM agents in a psychiatric emergency room. For ziprasidone, the dosage was 20 mg for 23 episodes and 10 mg for 5 episodes. For 24 episodes treated with combined haloperidol and lorazepam, the dosages were 4.8 ± 0.3 SEM mg and 1.9 ± 0.4 mg respectively. Outcomes were the duration of restraint and need for adjunctive "rescue" medications within 60 minutes. These outcomes were decided prior to reviewing any records.

**Results:**

No difference was found in restraint duration (ziprasidone, N = 28, 55 ± 5 minutes; haloperidol with lorazepam N = 24, 65 ± 7 minutes, P = NS). Use of "rescue" medications did not differ between the two groups. No changes in blood pressure were found, but pulse decreased 8.3 ± 2.4 for haloperidol with lorazepam and 8.9 ± 4.24 for ziprasidone (P = NS). No instances of excessive sedation or extra-pyramidal symptoms were documented.

**Conclusion:**

In this study, IM ziprasidone appeared effective, well tolerated, and similar in clinical profile to combined conventional IM medications for treating severe agitation in adolescents. Given the reportedly favorable acute side effect profile of parenteral atypical agents, they may provide an alternative to conventional antipsychotics for treating acute agitation in both adult and adolescent populations. Future randomized, controlled studies are needed.

## Background

Patients with agitation commonly present to psychiatric emergency services [PES]. Sedatives and mechanical restraints have been mainstays of treatment for severe agitation. As use of restraints is under increasing scrutiny because of potential increased morbidity and mortality, rapid and effective pharmacological management of severe agitation is critical [1].

Oral medication can be impractical or impossible in severe agitation. Conventional IM first-generation antipsychotics given as monotherapy or combined with lorazepam are commonly used. These agents may be associated with adverse effects, e.g. dystonia and other extra-pyramidal symptoms with haloperidol, and excess sedation with benzodiazepines [2].

Atypical antipsychotics have gained acceptance as first-line treatment for psychotic disorders. These agents have shown greater efficacy in some studies and a more favorable acute side effect profile. Currently, risperidone is indicated for the treatment of schizophrenia in adolescents age 13–17 years, bipolar mania in children and adolescents age 10–17 years, and irritability associated with autistic disorder in children and adolescents age 5–16. Aripiprazole is indicated for schizophrenia in children and adolescents age 13–17 years and bipolar mania in children and adolescents age 10–17 years. Several recent studies have described results of oral atypical antipsychotics in pediatric populations with a variety of diagnoses, e.g., ziprasidone for Tourette's disorder [3], risperidone for autistic disorder [4], olanzapine for bipolar mania [5], and aripiprazole for schizophrenia [6]. In general, these studies show positive clinical benefits.

Based on a combination of evidence- and consensus-based methodologies, a recent expert panel addressing treatment in children and adolescents recommended an expanded role for atypical antipsychotics, stating "Recommendation 5: Use an atypical antipsychotic first rather than a typical antipsychotic to treat aggression" [7]. The recommendation specifically included a broader range of diagnoses stating, "When psychosocial and first-line medication treatments for primary non-psychotic conditions have failed, physicians initially should use first-line atypical (rather than typical) antipsychotic medications to treat severe and persistent aggression" (Pappadopulos et al., 2003, p.151). The rationale for recommending atypical antipsychotics was principally their more favorable acute side-effect profile compared to older agents such as haloperidol (when used alone).

Ziprasidone was the first atypical antipsychotic agent available in parenteral form. In clinical trials for agitation in adults with psychotic disorders, it showed clinical effect within 15–30 minutes [8,9]. It was well tolerated with a low incidence of dystonia and EPS, and not found to produce oversedation. Despite a more favorable acute side-effect profile compared to older agents, it is associated with risk of QT_C _prolongation and a potential for arrhythmias [10]. Clinical trials of IM ziprasidone excluded children and adolescents, patients with severe agitation, and those whose agitation was associated with substance abuse.

Evidence is emerging from some studies that parenteral atypical antipsychotics may be useful in treating children and adolescents. Four retrospective studies have been published on IM ziprasidone for agitated adolescents [11-14], but none were conducted in a PES setting or compared ziprasidone to haloperidol. All four studies indicated positive clinical benefits without serious adverse effects.

As part of a naturalistic, retrospective observational study of IM ziprasidone versus conventional IM sedatives in agitated patients in the PES at SUNY Stony Brook, we obtained data on 110 adults receiving IM ziprasidone [15,16]. We found IM ziprasidone worked more quickly than in the published clinical trials, and that is was effective for agitation associated with substance abuse. During this naturalistic, retrospective observational study, we also treated 28 adolescents with IM ziprasidone and 24 adolescents with conventional IM sedatives.

## Methods

### PES Background

The SUNY Stony Brook PES receives ~6800 cases annually. Approximately 80% of patients present with major psychiatric illness and/or alcohol- or substance-induced intoxication. Approximately 60% of patients arrive by police escort, and 40% are involuntarily admitted after evaluation. Approximately 50% of patients receive medication. About 20% of referred cases are children or adolescents (age <18).

### Study Design

This was a retrospective, naturalistic outcome, nonrandom study (choice of sedative drug was by clinician preference). The study was approved by the institutional human subjects committee.

### Patients

A search of consecutive restraint records for episodes of agitation revealed 76 adolescents (age 12–17 years old) received an IM medication at the time of restraint, from October 2002 to August 2006. Of these, 24 were excluded: 4 who received ziprasidone with lorazepam, 6 who received oral or IM sedatives within 1 hour prior, and 14 who received miscellaneous IM agents (diphenhydramine, lorazepam, amobarbital, or chlorpromazine). No records were found for IM haloperidol monotherapy.

Demographic features of the resulting 2 groups are shown in Table 1. The most common primary clinical diagnoses were substance related disorders (N = 7), psychotic disorders (N = 7), adjustment disorders (N = 7), and impulse control disorders (N = 5). Of the 52 patients, 15 were on no home medications, while 26 were prescribed atypical antipsychotics, 21 mood stabilizers, 19 antidepressants, 9 stimulants, and 4 miscellaneous. These home medication types did not differ by group. Positive toxicology was found in 17/52 cases; the most common agents were cannabis (N = 14), benzodiazepines (N = 5), and cocaine (N = 4). There was no difference in the hospitalization rate for the 2 groups. In all cases the episode of agitation resolved. The patients were ambulatory at the time of discharge from the PES or transfer to a psychiatric hospital.

**Table 1:** Sample characteristics.

Characteristic	Ziprasidone (N = 28)	Haloperidol + Lorazepam (N = 24)	P
Age (years) [SD]	15.5 ± 1.5	15.9 ± 1.2	NS
Gender male/female	12/16	15/9	NS
Police Escort*	16/28	17/24	NS
Toxicology Positive**	7/28	10/24	NS
Psychiatrically Hospitalized	8/28	8/24	NS

*Police Escort = Adolescent was brought to hospital and escorted to PES by police due to violent or uncontrollable behavior.

**Urine toxicologies were often positive for multiple substances. Most common substances were: cannabis N = 14, benzodiazepines N = 5, and cocaine N = 4.

### Treatment

Adolescents had received either a single dose of ziprasidone 10 mg IM (N = 4) or ziprasidone 20 mg IM (N = 24) or IM haloperidol (average dose = 4.8 ± 0.3 mg, range 2.5–10), the latter combined in all cases with IM lorazepam (average dose = 1.9 ± 0.4 mg, range 1–2) (N = 24). Restrained patients received medication simultaneously with initiation of physical restraint. Any additional oral or parenteral sedative given within the next 1 hour was considered a rescue medication. Duration of restraint episodes was obtained from nursing documentation in the progress notes or the restraint records.

### Assessments

**Figure 1:**
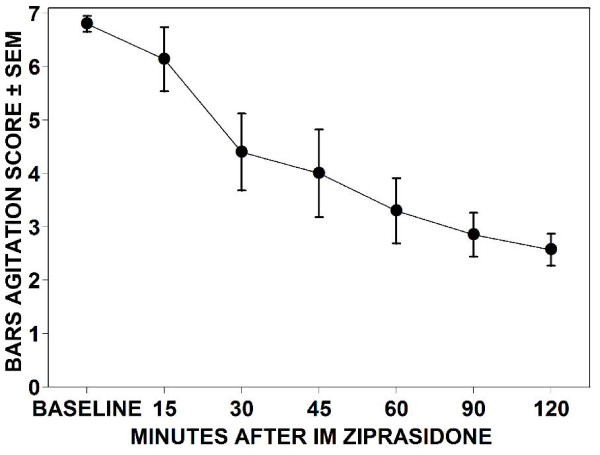
**Mean Behavioural Activity Rating Scale (BARS)**. (1 = difficult or unable to arouse; 2 = asleep but responds normally to verbal or physical contact; 3 = drowsy, appears sedated; 4 = quiet and awake [normal level of activity]; 5 = signs of overt [physical or verbal] activity, calms down with instructions; 6 = extremely or continuously active, not requiring restraint; 7 = violent, requires restraint) scores for 7 adolescent patients after treatment with IM ziprasidone. Scores at 30 minutes (p < 0.05) and thereafter (p < 0.01) were significantly different from baseline (Tukey test).

Concurrent agitation scores using the Behavioral Activity Rating Scale (BARS [17], Figure 1) recorded every 15 minutes from baseline to 120 minutes were available for 7 of the ziprasidone subjects and none of the comparison group. Scores on the BARS range from 1 to 7, where 1 = difficult or unable to arouse, 2 = asleep but responds normally to verbal or physical contact, 3 = drowsy, appears sedated, 4 = quiet and awake (normal level of activity), 5 = signs of overt (physical or verbal) activity, calms down with instructions, 6 = extremely or continuously active, not requiring restraint, and 7 = violent, requires restraint.

### Data Analyses

Comparisons were made by t-test, ANOVA using repeated measures technique with Tukey's comparison tests, and the chi-squared test.

## Results

### Outcomes

Baseline BARS scores for a subset of adolescents receiving ziprasidone were initially high and decreased rapidly. A significant decrease was found at 30 minutes and thereafter. The initial scores and time course after treatment were not different from agitated adults in our PES study (Figure 1).

**Figure 2:**
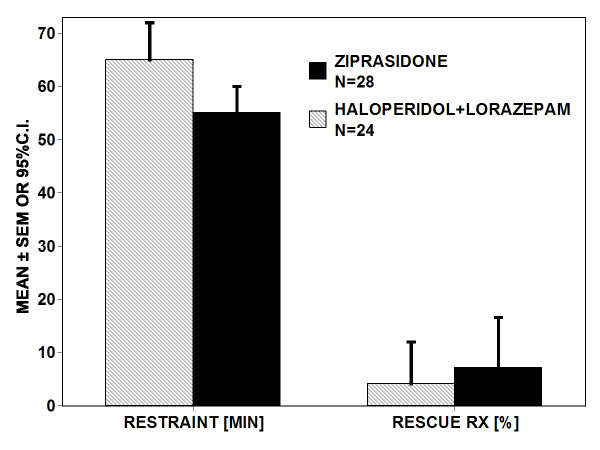
**No difference was found in restraint duration**. (ziprasidone, N = 28, 55 ± 5 minutes; haloperidol combined with lorazepam, N = 24, 66 ± 7; P = NS). Use of rescue medications did not differ between ziprasidone (2/28) and haloperidol combined with lorazepam (1/24).

No difference was found in restraint duration (ziprasidone N = 28, 55 ± 5 minutes; haloperidol with lorazepam N = 24, 65 ± 7 minutes, P = NS) as shown in Figure 2. Use of rescue medications did not differ between ziprasidone (2/28) and haloperidol combined with lorazepam (1/24) (χ^2 ^= 0.2, P = 0.6).

**Figure 3:**
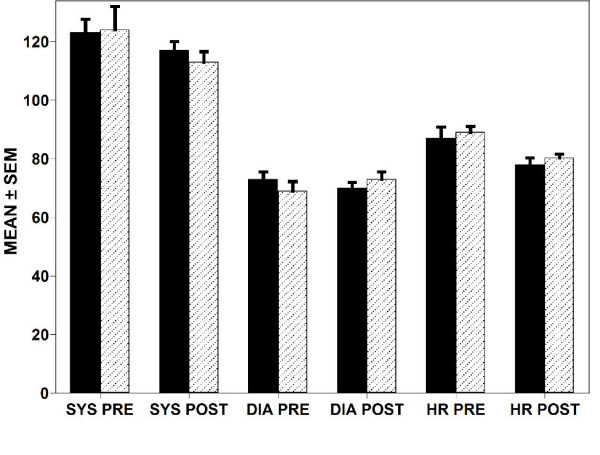
**Blood pressure/heart rate data were available for 18 patients before and after ziprasidone IM and 12 for combined conventional agents**. No overall pre-post changes in blood pressure were found, but pulse decreased 8.3 ± 2.4 for haloperidol combined with lorazepam and 8.9 ± 4.24 for ziprasidone (P = NS). SYS = systolic blood pressure; DIA = diastolic blood pressure; HR = heart rate; PRE = prior to treatment with IM medication; POST = after treatment with IM medication.

Ziprasidone IM was well tolerated in adolescents as shown in Figure 3. For adolescents, usable blood pressure and heart rate data were available for 18 patients before and after ziprasidone IM and 12 for combined conventional agents. No overall pre-post changes in blood pressure were found, but pulse decreased 8.3 ± 2.4 for haloperidol combined with lorazepam and 8.9 ± 4.24 for ziprasidone (P = NS) (figure 3). Of 28 ziprasidone cases, 4 post treatment ECGs were available and showed normal QTc (387–451 milliseconds). There were no recorded extrapyramidal reactions or administrations of anticholinergic agents.

## Discussion

The results of this naturalistic, retrospective, observational study suggest that IM ziprasidone monotherapy may be as effective for the treatment of severe agitation as combined IM conventional agents in severely agitated adolescents presenting to a PES. There was no difference in the duration of restraints or need for rescue medication between the two groups. In addition, among 7 unselected episodes of agitation that were rated on the BARS, there was a rapid and significant improvement within 30 minutes from initial high severity scores. Future randomized as well as observational trials will show whether monotherapy with ziprasidone has similar efficacy to the combination of conventional agents haloperidol with lorazepam. However, consent issues will make prospective trials difficult.

The data represent a naturalistic outcome of unselected cases that were typical of severely agitated adolescents who presented to a PES. We defined severe agitation as episodes requiring physical restraint. Cases were identified by searching restraint logs. Hence, cases of IM medication use not requiring restraints were not captured. Although the samples represented a consecutive case series, the patients were not randomly assigned to treatment, and the choice of an IM agent for agitation was not controlled. The possibility of missing documentation of adverse drug events in this study prevents definitive conclusions regarding the safety of these agents in children and adolescents. Thus the results of this study are tentative due to the possible methodological limitations, including potential retrospective biases, lack of more extensive results from the standardized rating scale of agitation, and modest sample size.

Although the two treatment groups were identified retrospectively, the 28 ziprasidone-treated adolescent patients in the present analysis were similar in demographics, had similar proportions of need for rescue medication, restraint use, and duration of restraint compared to the 24 subjects in the comparison group who received conventional IM agents. These observations suggest that there is no apparently systematic difference between the two final groups that would have biased outcome after parenteral medication. However, as previously stated, modest sample size may have created false negative findings in regard to demographic information.

For the subgroup of 7 patients rated on the BARS, symptoms of severe agitation were significantly reduced within 30 minutes after a single dose of IM ziprasidone, for up to 120 minutes. The mean baseline BARS score of 6.9, although high, was not statistically different from that (6.6 ± 0.1) in the entire adult population of agitated patients (N = 110) in the original study as well as in a sub-sample of geriatric agitated patients (6.8 ± 0.1) [15,16]. These scores are similar to those reported by retrospective ratings of 59 agitated adolescents treated with parenteral ziprasidone whose scores were 6.5 ± 0.7 [11]. The high BARS scores in these naturalistic samples suggest that these studies show more severe agitation than the published clinical trials of intramuscular ziprasidone for adults with schizophrenia where the mean BARS score was 5.0 [18]. The agitation scores for adolescents showed similar rates of clinically and statistically significant reduction at 45 minutes after ziprasidone administration (-42%), as did the adults in our prior study (-50%). The BARS is not part of routine care. The 7 patients for whom BARS data was available had participated in a prior study [15]

Atypical antipsychotics in the oral formulation have gained acceptance as first-line treatments for psychosis. Overall, the atypical neuroleptics have been shown to have similar effectiveness in available studies and to have a more favorable acute adverse-effect profile compared with first-generation antipsychotic agents. Some of these agents may pose a greater risk for early weight gain and metabolic consequences when used for ongoing treatment. Few studies of the effects of IM neuroleptics in the adolescent population are available. Pending randomized controlled trials, which are not likely to be readily available for severe agitation in adolescents, this study and prior studies suggest that parenteral atypical antipsychotics may be useful for short-term treatment of severe agitation in this clinical setting. Oral antipsychotic use after IM antipsychotic use was not captured in this study, and so issues regarding transition to oral medication cannot be addressed.

## Conclusion

The results of this study indicate that reduction in severe agitation in the ziprasidone IM monotherapy group was comparable to the haloperidol IM combined with lorazepam IM group. Clinical outcomes were similar for time in restraint and need for rescue medications. The results of this study are tentative but consistent with the prior literature that ziprasidone IM is safe and well tolerated in adolescents. IM atypical antipsychotics such as ziprasidone offer an emerging alternative to butyrophenones, benzodiazepines, or their combination for management of severe agitation.

## Competing interests

Dr. Preval is on the speaker's bureau for Pfizer and Bristol Myers Squibb-Otsuka. Mr. Southard is on the speaker's bureau for Janssen, Astra-Zeneca, and Bristol Myers Squibb-Otsuka and formerly for Pfizer. Dr. Klotz is currently a speaker for Pfizer and Eli Lilly; previously he has spoken for or consulted to Bristol Myers Squibb-Otsuka and Wyeth. Dr. Francis was formerly a consultant for Pfizer. Dr. Jangro has no conflicts of interest or financial ties to disclose.

## Authors' contributions

WJ participated in the coordination and design of the study, collected data, and drafted the manuscript. HP conceived of the study, and participated in its design and coordination. RS collected data. SK collected data. AF collected data, drafted the manuscript, conceived of the study, and participated in its design and coordination. All authors read and approved the final manuscript.
